# Have you tried turning it off and on again? Oscillating selection to enhance fitness-landscape traversal in adaptive laboratory evolution experiments

**DOI:** 10.1016/j.mec.2023.e00227

**Published:** 2023-07-13

**Authors:** Alexander C. Carpenter, Adam M. Feist, Fergus S.M. Harrison, Ian T. Paulsen, Thomas C. Williams

**Affiliations:** a Department of Molecular Sciences and ARC Centre of Excellence in Synthetic Biology, Centre Headquarters, Macquarie University, Sydney, SW, 2109, Australia; b CSIRO Synthetic Biology Future Science Platform, Canberra, ACT, 2601, Australia; c Department of Bioengineering, University of California San Diego, 9500 Gilman Dr., La Jolla, CA, 92093, USA; d Joint BioEnergy Institute, 5885 Hollis Street, 4th Floor, Emeryville, CA, 94608, USA; e Novo Nordisk Foundation Center for Biosustainability, Technical University of Denmark, 2800, Kgs, Lyngby, Denmark

## Abstract

Adaptive Laboratory Evolution (ALE) is a powerful tool for engineering and understanding microbial physiology. ALE relies on the selection and enrichment of mutations that enable survival or faster growth under a selective condition imposed by the experimental setup. Phenotypic fitness landscapes are often underpinned by complex genotypes involving multiple genes, with combinatorial positive and negative effects on fitness. Such genotype relationships result in mutational fitness landscapes with multiple local fitness maxima and valleys. Traversing local maxima to find a global maximum often requires an individual or sub-population of cells to traverse fitness valleys. Traversing involves gaining mutations that are not adaptive for a given local maximum but are necessary to ‘peak shift’ to another local maximum, or eventually a global maximum. Despite these relatively well understood evolutionary principles, and the combinatorial genotypes that underlie most metabolic phenotypes, the majority of applied ALE experiments are conducted using constant selection pressures. The use of constant pressure can result in populations becoming trapped within local maxima, and often precludes the attainment of optimum phenotypes associated with global maxima. Here, we argue that oscillating selection pressures is an easily accessible mechanism for traversing fitness landscapes in ALE experiments, and provide theoretical and practical frameworks for implementation.

## Highlights

• ALE is a powerful tool for engineering and understanding microorganisms.
• Constant selection pressure prevents navigation of rough fitness landscapes.
• Oscillating selective pressure can enable fitness valley crossing and peak shifting.
• These approaches will increase the chances of reaching global maxima in ALE.

## 1 Microbial cell factories

Microbial fermentation has emerged as a sustainable alternative for manufacturing of industrial, medical, and agricultural products. In these applications, microbial metabolism is harnessed to convert renewable or waste carbon and energy into valuable products such as chemicals, fuels, foods, materials, and pharmaceuticals. While there have been tremendous successes from traditional metabolic engineering efforts, it is widely recognised that the pace of innovation is too costly and too slow (Nielsen and Keasling Jay, 2016). These limitations arise due to the highly complex nature of biological systems, their partial characterisation, and their evolutionarily optimisation for proliferation and survival under diverse conditions (Nielsen and Keasling Jay, 2016).

The sometimes-overwhelming complexity of biological systems has led to the rise of semi-rational design and high-throughput screening in biofoundries, where thousands of genetic variants are designed, built, and tested for a target phenotype (Marcellin and Nielsen, 2018). Despite these approaches having great utility, they fall well short of addressing the available biological design space for a given phenotype, which is typically underpinned by millions of potential genotypic variations. Furthermore, there are biological ‘unknown unknowns’ that can only be uncovered by a truly untargeted process capable of screening hundreds of millions of variants. Adaptive Laboratory Evolution (ALE) has emerged as a tool with the potential to address all these problems through the optimisation of microbial phenotypes using evolution (Sandberg et al., 2019).

## 2 Adaptive laboratory evolution

**Fig. 1:**
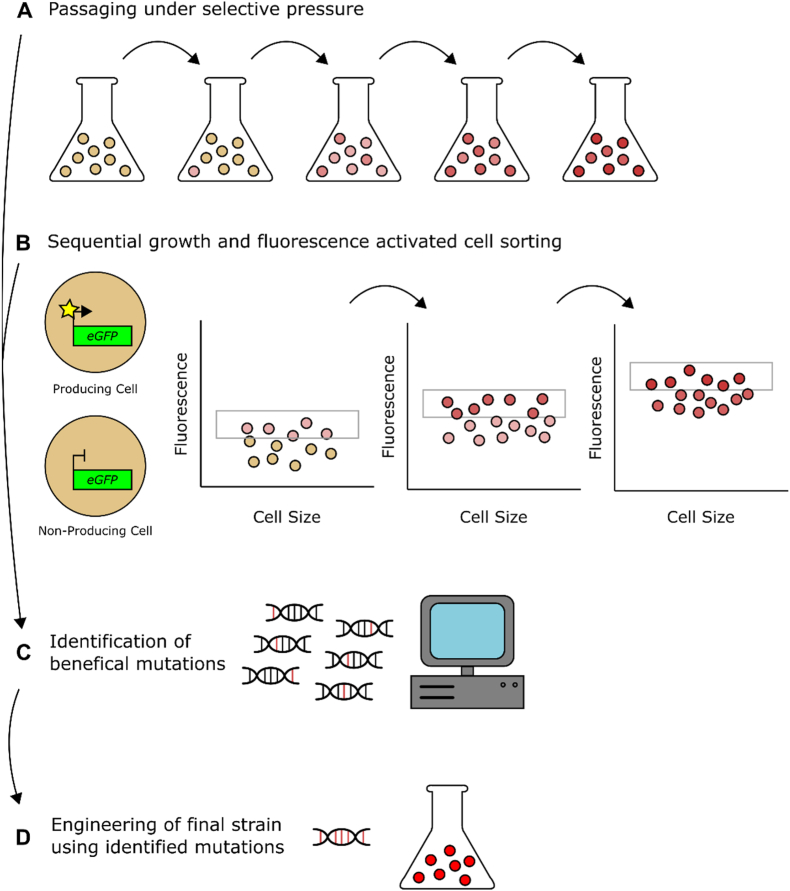
**Adaptive laboratory evolution work-flow** **A)** Passaging of cells under selective pressure selects for cells with increased fitness (coloured red). Continual selection enriches higher performing variants in the population. **B)** A biosensor which increases fluorescence in response to a compound of interest can be used to identify cells with increased production (coloured red). Continual growth and fluorescence activated cells sorting (FACS) increases the proportion of higher producing members in the population. **C)** A combination of genomics and systems biology is used to identify beneficial mutations. **D)** Beneficial genetic variants are reverse engineered into the parental strain to elucidate genotype-phenotype relationships and to increase production/survival/growth. (For interpretation of the references to colour in this figure legend, the reader is referred to the Web version of this article.)

ALE is a process in which organisms are subjected to an environment which offers a selective advantage to cells which perform a desired function (LaCroix et al., 2017; Dragosits and Mattanovich, 2013; Mans et al., 2018). Over time, cells which possess advantageous mutations relevant to the selective pressure have an increased chance of becoming dominant within the population (Fig. 1) (LaCroix et al., 2017; Dragosits and Mattanovich, 2013; Mans et al., 2018; Portnoy et al., 2011; Shepelin et al., 2018). It is a semi-continual process that can be executed over a time scale of weeks to years (Dragosits and Mattanovich, 2013). Traditionally, ALE has been used to evolve phenotypes with inherent selective advantages, where beneficial mutations directly aid the cell's ability to grow and reproduce (Dragosits and Mattanovich, 2013; Mans et al., 2018; Portnoy et al., 2011; Shepelin et al., 2018). For example, evolution of alternative substrate utilization, resistance to toxic compounds, and increased temperature tolerance (Ho et al., 2017; Caspeta et al., 2014; Voordeckers et al., 2015; Espinosa et al., 2020; Meyer et al., 2018; Xu et al., 2019).

In recent years, a new focus of ALE has emerged in which phenotypes with no inherent growth advantage are being targeted for selection. That is, metabolite production phenotypes which offer no reproductive advantage are increasingly the focus of ALE experiments. While it is sometimes possible to engineer these selective pressures using gene knockouts that couple desired carbon fluxes to growth, these mechanisms are highly context specific and not sufficiently universal for broad application to most metabolic pathways (Alter and Ebert, 2019). ALE of metabolite production phenotypes is therefore increasingly being investigated using biosensors (Williams et al., 2016). Biosensors are commonly proteins, nucleic acids, or genetic circuits that can convert the presence of a chosen metabolite into a change in gene expression/measurable signal that the researcher can use to apply a selection pressure (Carpenter et al., 2018). The output of such a biosensor could be the complementation of an auxotrophic marker, or the production of a fluorescent protein, allowing selection with growth or fluorescent activated cell sorting (FACS), respectively. This allows the expansion of the types of phenotypes that can be selected for in ALE as they allow the coupling of production of non-essential metabolites into a selectable output. However, FACS-based screening does have a reduced throughput compared to growth-based selection, with FACS screening 10–100 million cells per day, compared to growth based selection of 10–100 million cells per mL of liquid culture (Dragosits and Mattanovich, 2013; Sciambi and Abate, 2015). Whilst not as common as ALE using growth-coupled outputs, FACS-based selection and high-throughput screening has been used for improved production of a variety of compounds including 3,4 dihydroxy benzoate, phenylalanine, threonine, and 1-butanol (Jha et al., 2014; Liu et al., 2015, 2017; Dietrich et al., 2013).

## 3 Constant selection pressure

**Fig. 2:**
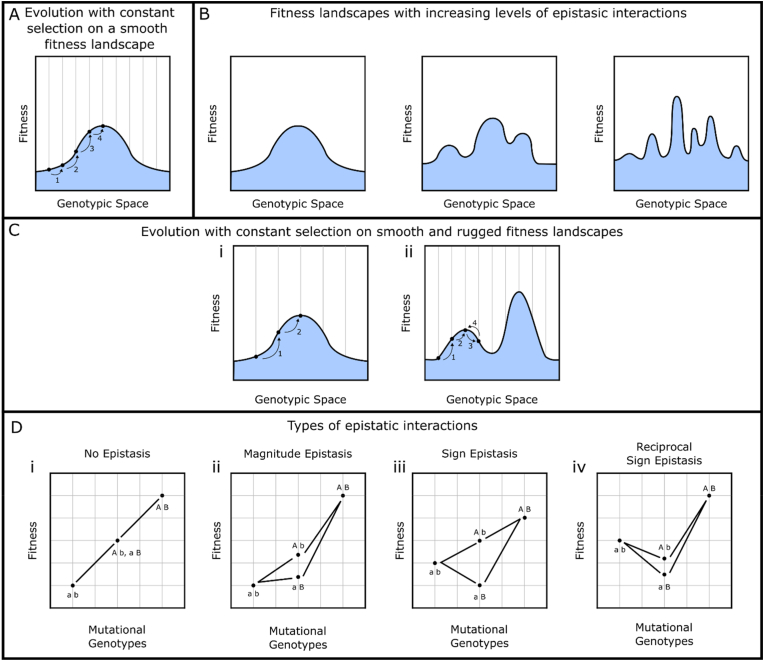
**Effects of epistatic interactions on evolutionary trajectoriesA**) A smooth fitness landscape demonstrating the stepwise evolution of a population to a higher fitness state by progressive mutations. As cells with beneficial mutations arise their increased fitness allows easier fixation within the population. This can occur sequentially, raising the total fitness. **B**) A series of fitness landscapes progressing from smooth to rugged (left to right). The smooth fitness landscape is defined by a single peak which is approachable via gentle slopes. The more rugged fitness landscapes possess multiple peaks of various heights, separated by valleys of lower fitness. **C**) **i)** Evolution on a smooth fitness landscape with a constant selection pressure is straightforward. Cells with beneficial mutations that arise in the population can sequentially fixate in a series of steps that follow and uphill progression towards the global peak. **ii)** Evolution on a rugged fitness landscape with a constant selection pressure is less easy. The population is able to climb to the smaller peak in the same way as previously described. However, movement from the smaller peak to the larger requires cells to possess deleterious mutations. The constant selection pressure drives cells that might cross the valley back to the local-optimum fitness peak. **D**) Relative fitness of simplified genotype combinations demonstrating no epistasis, magnitude epistasis, sign epistasis, and reciprocal sign epistasis. Genomes are wildtype (i.e., a,b) with mutations A or B or both. **i)** Under no epistasis both mutations contribute in an additive fashion to fitness, where the double mutant has the sum fitness of each mutation alone. **ii)** Under magnitude epistasis the effect of each mutation in isolation is less than the double mutant. The effect on fitness of the double mutant is greater than the sum of the individual mutants. **iii)** Under sign epistasis one of the mutations has a negative effect on fitness when it occurs in isolation, but a positive effect when the other mutation is present. **iv)** Under reciprocal sign epistasis both mutants have a negative effect of fitness when they occur in isolation, but together increase fitness.

Selective pressures in ALE experiments are largely applied either as a constant force or a constantly increasing force (Dragosits and Mattanovich, 2013; Mans et al., 2018; Shepelin et al., 2018). In each round of growth and selection, mutations can occur through natural mechanisms or induced via mutagenesis, and the fittest members of the population taken on to subsequent rounds (LaCroix et al., 2017; Dragosits and Mattanovich, 2013). For growth-based selection schemes this can be as simple as passaging a portion of the population into new media, and for fluorescence-based schemes, using FACS to isolate the brightest cells (Schallmey et al., 2014). The percentage of the population that is passaged varies based on experimental design and often depends on the time and resources available. Studies have suggested a range of population percentages to passage between rounds, ranging from 1% to 13.5%–20% citing trade-offs between evolutionary bottle-necking and the time available (LaCroix et al., 2017; Wahl and Gerrish, 2001; Hubbarde and Wahl, 2008). Regardless of the percentage passaged, the concept is that the average fitness of the population will slowly “walk” up a fitness landscape (Fig. 2A). This process of passaging under selection can be iterated upon until improvements in fitness plateau.

## 4 Fitness landscapes and epistasis

A constant selection strategy is most applicable under the assumption that the fitness landscape to be traversed is relatively smooth. Smooth landscapes allow the accumulation of mutations in a stepwise manner, where each new selected mutation increases fitness. The mutations involved in the phenotype do not need to occur in a specific order as each mutation has an additive effect. Constant selection is used frequently for directed evolution of proteins (Romero and Arnold, 2009; Tracewell and Arnold, 2009). However, a constant selection strategy becomes less efficient, or even completely ineffective, when the fitness landscape to be traversed is more rugged (Barton, 2017; Ochs and Desai, 2015). In contrast to smooth landscapes which possess generally positive gradients towards a global peak, rugged landscapes contain multiple lower peaks separated by valleys of reduced fitness (Barton, 2017; Ochs and Desai, 2015; Obolski et al., 2017) (Fig. 2B). Constant positive selection tends to drive the population up the genetically closest fitness peak (de Vos et al., 2015). However, rather than being the global maximum, this peak may only be a local maximum. Once the population has been driven to a local maximum, the constant selective pressure applied to the system can make it difficult for the population to explore other peaks within the fitness landscape (Fig. 2C) (Sailer and Harms, 2017; Weissman et al., 2009; Steinberg and Ostermeier, 2016). The ruggedness observed in fitness landscapes is due to epistatic interactions between mutations (Poelwijk et al., 2011).

## 5 Epistasis

Epistasis is the concept that the effect of a mutation on phenotype can be dependent on the genetic context in which that mutation occurs (Ochs and Desai, 2015; Obolski et al., 2017). Some mutations which may be important for reaching a fitness maximum may have a neutral, negative, or diminished effect if a previous mutation is not present (Barton, 2017; Sailer and Harms, 2017; Weissman et al., 2009; Van Cleve and Weissman, 2015). A hypothetical example of this is the evolution of ethanol production. Ethanol production can garner a selective advantage to cells as a defence mechanism in mixed populations, but only if they have a mechanism to resist the ethanol they are producing. A cell which evolved increased ethanol production without evolving tolerance would be at a disadvantage relative to the rest of the population. The three most relevant types of epistasis to this article are magnitude, sign, and reciprocal sign (Fig. 2D) (Obolski et al., 2017; Poelwijk et al., 2007; Dawid et al., 2010). Magnitude epistasis occurs when the size of a phenotypic effect resulting from the combination of two or more mutations does not equal the size of effects observed for each mutation in isolation. Rather than each mutation contributing to the phenotypic effect in an additive fashion, the net change in phenotype is greater than the sum of its parts (Poelwijk et al., 2007; Weinreich et al., 2005). Sign epistasis is when the effect a mutation has on fitness is inverted from advantageous to deleterious or *vice versa*, depending on the genetic context in which it occurs (Poelwijk et al., 2007; Weinreich et al., 2005). That is, a mutation may be deleterious in isolation, but beneficial if a secondary mutation is also present. Finally, reciprocal sign epistasis occurs when two or more mutations improve fitness when all are present, but individually each mutation decreases fitness (Poelwijk et al., 2007).

Epistatic interactions are responsible for the rugged features of some adaptive landscapes. Specifically, sign epistatic interactions reduce the total number of viable trajectories, requiring mutations to arise in a specific order, or simultaneously (Sailer and Harms, 2017; Poelwijk et al., 2011). This has been exemplified in several protein evolution studies (Yang et al., 2019; Heckmann et al., 2013; Reetz and Sanchis, 2008; Sugrue et al., 2017). These studies reviewed the possible mutational trajectories which could be taken for a series of proteins, reconstructing and/or analysing the fitness of each possible mutant within the fitness landscape. Each found that sign epistasis restricted the possible routes of evolution under a constant selection regime (Yang et al., 2019; Reetz and Sanchis, 2008; Sugrue et al., 2017). Alternatively, reciprocal sign interactions create fitness peaks and valleys (Barton, 2017; Kvitek and Sherlock, 2011; Chiotti et al., 2014). Rather than restricting evolutionary trajectories to a specific subset of mutations, reciprocal sign interactions do not offer an uphill path to the global maximum, trapping populations on local peaks. Metabolism is an extremely complex network including many feedback, inhibitory, synergistic, and antagonistic relationships both at the transcription/translational and enzyme kinetics levels (Alam et al., 2016; Sato et al., 2016). As such, the level of epistatic interactions within the system are quite high (Snitkin and Segrè, 2011; Szendro et al., 2013; Weinreich et al., 2013). Despite this, most ALE experiments still operate with selection schemes that are optimized for evolution on smooth fitness landscapes. These selection schemes struggle to tackle the unique challenges posed by rugged landscapes and entrapment on local peaks. This was highlighted by Kvitek and Sherlock (2011) in a study in which 448 generations of *Saccharomyces cerevisiae* were grown on glucose limiting conditions. This resulted in the identification of adaptive mutations in *MTH1* and *HXT6/*7 that occurred in separate lineages but not simultaneously. Further investigation revealed that due to epistatic interactions, cells containing both mutants suffered a significant fitness penalty. Had the experiment been initiated with one of the numerous *S. cerevisiae* strains which naturally possess the *MTH1* mutation, the probability of identifying the *HXT6/7* variation would have been severely reduced (Kvitek and Sherlock, 2011).

## 6 Stochastic tunnelling/shifting balance of evolution

**Fig. 3:**
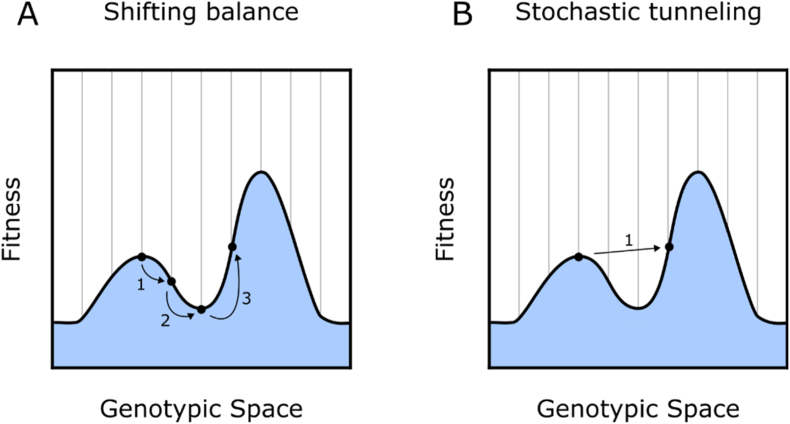
**Valley crossing under shifting balance and stochastic tunnelling theories of evolution** Simplified evolutionary trajectories for shifting balance and stochastic tunnelling theories of valley traversal. **A)** In shifting balance theory the population under consideration needs to be sufficiently small such that genetic drift is capable of overpowering selection. In this example genetic drift has allowed the fixation of an intermediate genotype despite its negative effect on fitness **B)** Stochastic tunnelling theory suggests that members within a population may develop intermediate deleterious genotypes followed by additional beneficial mutations before the effect of selection drives the intermediate genotypes to extinction. This allows for valley crossing without fixation of less fit genotypes.

The main challenges for ALE on rugged landscapes involve peak navigation. Specifically, the avoidance of local maximums and the movement from one peak onto adjacent peaks, whilst crossing fitness valleys. There is a large quantity of theoretical and experimental research that has focused on evolutionary peak shifts via valley crossings, which is covered in several excellent studies (Barton, 2017; Ochs and Desai, 2015; Obolski et al., 2017; Weissman et al., 2009; Poelwijk et al., 2007). Whilst research in this area is still on going, and multiple hypotheses exists, the two classic theories of how populations cross fitness valleys are shifting balance theory (SBT) and stochastic tunnelling (Fig. 3) (Iwasa et al., 2004; Wright, 1932). SBT suggests that if populations are divided into smaller groups, the contribution of genetic drift will be much higher and the likelihood of non-adaptive mutations becoming fixed increases. Thus, a subdivided population could stochastically fix mutations that correspond with fitness valleys. This subdivided population would then be capable of acquiring new mutations which place it in an adjacent fitness peak. Alternatively, stochastic tunnelling suggests that a small number of cells within a population may be able to accrue sufficient mutations to move from one peak to another without fixating the intermediate genotypes. That is, some cells within the population may develop deleterious mutations, but before becoming extinct develop additional mutations which place them in an adjacent fitness peak. From an ALE perspective, neither of these theories offer quick escape routes from local maxima, with even simple peak shifts sometimes requiring millions of generations (Ochs and Desai, 2015; Obolski et al., 2017; Weissman et al., 2009).

## 7 Varying environments for valley crossing

Since the establishment of SBT and stochastic tunnelling, other possible mechanisms of valley crossing have also been investigated with promising biotechnological applications. One such mechanism is that of environmental fluctuation. Adaptive landscapes are selection pressure specific, with different environments favouring different evolutionary trajectories (Taute et al., 2014; Pál and Papp, 2017). That is, a genotype with high fitness under one set of conditions may not necessarily have high fitness under a separate set of conditions. The key obstacle in fitness valley crossing is how intermediate deleterious genotypes can survive selection and allow escape from a local maximum (Obolski et al., 2017; Pál and Papp, 2017). It has been suggested that by altering the nature of the selection pressure, these intermediate genotypes could become neutral or even advantageous under the new fitness landscape (Pál and Papp, 2017; Hadany, 2003; Flynn et al., 2013). Thus, the alteration of selective pressure allows the exploration of mutations that would be neutral/deleterious in other environments, expanding the number of unique trajectories which can be explored, and allowing cells which have reached a local maximum an opportunity to “step off” that peak (de Vos et al., 2015; Steinberg and Ostermeier, 2016; Taute et al., 2014; Hall et al., 2019; Szappanos et al., 2016; Kashtan et al., 2007). Rather than relying on rare simultaneous mutations which shift cells from one peak to another in a stochastic tunnelling event, altering selective pressure could stabilize an intermediate genotype in a manner not dis-similar to enzymes lowering the activation energy of a chemical reaction. Given the epistatic nature of many phenotypes relevant to metabolic engineering such as metabolic flux, enzyme structure and activity, and transcriptional regulation, we argue that oscillating selective conditions can be employed in ALE experiments to enhance the performance of resulting isolates and populations (Snitkin and Segrè, 2011; Szendro et al., 2013; Weinreich et al., 2013).

## 8 Types of selection alteration

**Fig. 4:**
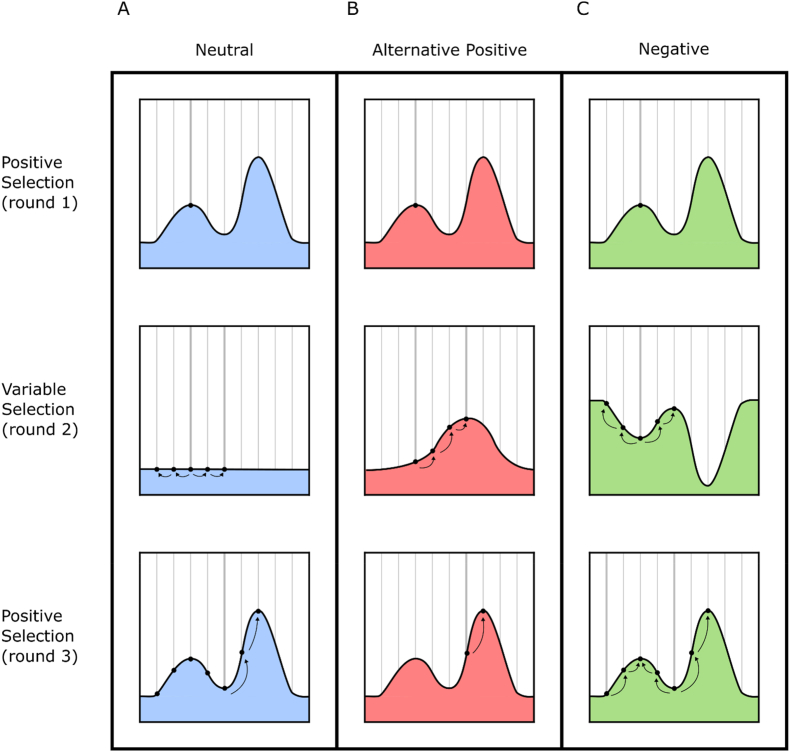
**Evolution on changing rugged fitness landscapes** Oscillation of selective pressure applied alters the fitness landscape of a simplified ALE experiment. The y axis on each graph is fitness and the x axis is genotypic space. Figure shows only three rounds of selection for clarity, however in practice multiple repeating rounds would be used. **A)** Oscillation with neutral selection imposes a flattening of the landscape, allowing the population to accrue non-beneficial mutations. When positive selection is resumed some members of the population may be able to ascend to the higher peak **B)** Oscillation with alternative positive selection imposes an alternative fitness landscape that may be similar to the original, but different enough to drive the population onto adjacent peaks. **C)** Oscillation with negative selection imposes a fitness landscape which is the inverse of the original. Selection in this manner drives cells away from the peak they were on in any direction which is available.

ALE methods that include selection pressure oscillation could enable the traversal through local maxima and enable the generation of higher performing strains. Several computational and physical experiments have indicated that varying selection pressure can assist in the evolution of desired traits (de Vos et al., 2015; Steinberg and Ostermeier, 2016; Szappanos et al., 2016; Kashtan et al., 2007; Maltas et al., 2021; Abdul-Rahman et al., 2021; Sandberg et al., 2017). Computational studies have examined slightly different aspects of selection alteration, with strategies for valley traversal falling into three broad main groups (Sandberg et al., 2019; de Vos et al., 2015; Steinberg and Ostermeier, 2016; Kashtan et al., 2007). Oscillating between positive selection and either neutral, alternative positive, or negative selection have all shown promise for fitness valley traversal (Fig. 4). Neutral selection in this context is the permissive growth/survival of the population independent of a directional selection. This would allow the majority of mutants that arise in the population to survive temporarily, giving cells the opportunity to “step off” a local maximum (Kashtan et al., 2007). Rather than relaxing selection, alternative positive selection involves changing to a secondary target of directed evolution that is likely to share sub-goals with the primary target. This would change the fitness landscape, possibly turning a genotype that was at a fitness peak to now be on a slope (de Vos et al., 2015; Szappanos et al., 2016). This allows constant positive selection, albeit with alternating targets. This was partially demonstrated by Abdul-Rahman et al. (Abdul-Rahman et al., 2021) in an experiment which analysed diversity in populations of *S. cerevisiae* passaged between two selective environments. In this study a library of 4000 mutants was grown in media that was carbon limited, nitrogen limited, or oscillated between the two conditions. By the conclusion of the experiment, both populations which had been grown under constant selection had simplified to the extent that a single mutant comprised more than 50% of their respective populations. This was in contrast to the population grown under oscillating conditions whose most dominant mutant made up only 3% of the population (Abdul-Rahman et al., 2021). The final and possibly most counter-intuitive oscillation strategy is negative selection, which involves actively selecting against the phenotype of interest. In this scenario, cells are actively selected to move away from the local maximum (Steinberg and Ostermeier, 2016). This technique relies on the concept that many routes are possible for descension and some of those routes may locate individuals at the base of an adjacent peak. This was highlighted by work conducted by Steinberg and Ostermeier (2016) which explored the evolution of β-lactam resistance using *Escherichia coli* populations expressing mutants of the β-lactamase gene *TEM-1*. This study incorporated negative selection by selecting cells with lower β-lactam resistance, which was then followed with positive selection by selecting cells with the highest β-lactam resistance. When compared to populations subjected to exclusively positive selection, the negative selection group was able to achieve a 4 fold higher increase in β-lactam resistance (Steinberg and Ostermeier, 2016).

## 9 Implementation in ALE experiments

Whilst the neutral, alternative positive, and negative methods of selective pressure oscillation have shown promise compared to constant selective pressure, questions remain as to which method is most effective, and how they can be most effectively implemented. Despite this, there are still actionable concepts that can be implemented in ALE experiments. Implementation of oscillation with neutral selection would be feasible for many ALE experiments by simply adding rounds of growth in permissive media whilst maintaining whichever method of genetic diversification is being used. The use of permissive growth has been used previously to aid the ALE of methanol utilization in *S. cerevisiae* by maintaining biomass between selection rounds (Espinosa et al., 2020). Oscillation with alternative positive selection could be implemented with alternative media for growth-based experiments, either by using a similar carbon source, stress inducing compound, or enzymatic substrate. This approach would become more difficult if a biosensor was used as the selection method, as a second biosensor for a closely related metabolite would be required. Additionally, oscillation with negative selection may prove challenging to implement depending on the selection regime used. ALE experiments investigating growth specific phenotypes would need rounds in which growing cells are actively selected against. Biosensor-based selection schemes would be easier to use with rounds of negative selection using counter-selectable markers or low fluorescence as negative selection of biosensor output.

The final challenges in implementing oscillating selective pressure in ALE experiments concern the stage and frequency of implementation. One might want to only begin oscillation once a plateau of fitness improvement is reached in a constant condition ALE experiment. This would allow for a low-risk method of exploring oscillating selection pressure and as a method to progress an otherwise successful, but plateaued experiment. However, it is plausible that for neutral and negative selection pressure oscillation, the further strains evolve into their local maximums, the harder it could be to leave that fitness peak. Additionally, data on the frequency with which selective pressure should be switched is sparse. However, some successful studies have previously changed selective pressure after 3–5 rounds of outgrowth on solid media, and 20 generations in liquid media (Steinberg and Ostermeier, 2016; Kashtan et al., 2007). It's conceivable that an overly high oscillation frequency would not provide sufficient opportunity for intermediate genotypes to arise before being lost to positive selection. Finally, consideration must be given to the overall selective pressure of the experiment. Experiments which use equal durations of positive and alternative selections provide no net fitness advantage to the desired phenotype. Rather than facilitating fitness-valley crossing, equi-duration oscillation may result in the evolution of distinct sub-populations (Sandberg et al., 2017). It is likely that the optimal oscillation frequency, duration, and stage of implementation will be dependent on several factors, including the organism's frequency of mutation, the level of sign epistasis within the system, and the genotypic distance between peaks.

## 10 Future outlook

The evolutionary trajectories of populations in ALE experiments are heavily impacted by experimental setup and the implementation of selective pressure. Many ALE experiments are conducted over the course of 100–500 generations or 1–2 months of daily passaging (Sandberg et al., 2019). While multiple mutations are typically present in the resulting populations and isolates, there is often a handful of simple causative mutations underlying the improved phenotype. This raises the question, is the lack of complex multigenic mutations identified by most ALE experiments a fundamental property of the phenotypes investigated, or an outcome of the experimental set-up? Given the clear complexity of most naturally evolved traits, we propose that the constant selective pressure typically used in ALE has contributed to the limited elucidation of complex traits. Additionally, as new forms of genetic diversification begin to be implemented in ALE experiments (e.g. SCRaMbLE, OrthoRep, MAGE etc), the ability to traverse rugged fitness landscapes will become more important (Wu et al., 2018; Rix et al., 2020; Wang et al., 2009). Historically ALE experiments have typically relied on mutation rates dictated by the natural DNA replication error rate of the species being evolved. This approach avoids the problem of advantageous and deleterious mutations arising in the same genome, but underexplores the genotypic solution space (Zheng et al., 2021). In contrast, mutagenesis methods relying on chemical mutagens and radiation can give rise to excessive mutation rates, with desirable mutations masked by deleterious ones (Lee et al., 2011). Further, safety concerns of such mutagenic compounds can limit their usability. However, an emerging set of synthetic biology approaches are aimed at increasing genetic diversity whilst decreasing the proportion of deleterious mutations. For example, techniques have been developed for controlling the *in vivo* DNA replication error rate either in response to the desired phenotype using a biosensor or for a selected DNA region (Rix et al., 2020; Chou and Keasling, 2013; Jensen et al., 2021). Additionally, efforts such as the synthetic yeast genome project (Sc 2.0) offer large scale rearrangements of entire transcriptional units (Annaluru et al., 2014). As these and other methods of genetic diversification become more commonplace in ALE experimentation, the nature of the mutational “step” will change, increasing the probability of escaping local fitness maximums. Combined with oscillating selective pressures, we predict this will enable more rapid traversal of rough fitness landscapes and allow an easier approach towards reaching global maxima.

## 11 Conclusions

Significant progress has been made in the field of evolutionary biology in understanding the processes that influence evolution of organisms in rugged fitness landscapes. The traditional view that evolution of complex traits must progress through slow/improbable mechanisms such as stochastic tunnelling or SBT is receiving considerable criticism (Obolski et al., 2017; Steinberg and Ostermeier, 2016). Selection pressure oscillation offers a viable method for improving evolutionary outcomes. However, the field of metabolic engineering has been slow to make use of these advances, despite the considerable improvement oscillation of selection could provide. Whilst optimum oscillation conditions have yet to be elucidated for the purposes of metabolic engineering, many opportunities exist for researchers to begin implementing versions of the technique into their work. This would allow ALE experiments to explore more diverse genetic space, decreasing the probability of entrapment on local fitness maximums, and increasing the probability of finding strains with improved target phenotypes.

## Declaration of competing interest

The authors declare the following financial interests/personal relationships which may be considered as potential competing interests: Both Alexander Carpenter and Thomas Williams have a financial interest in the company Number 8 Bio PTY LTD.

## Data Availability

No data was used for the research described in the article.

## References

[bib1] Abdul-Rahman F., Tranchina D., Gresham D. (2021). Fluctuating environments maintain genetic diversity through neutral fitness effects and balancing selection. Mol. Biol. Evol..

[bib2] Alam M.T., Zelezniak A., Mülleder M., Shliaha P., Schwarz R., Capuano F., Vowinckel J., Radmaneshfar E., Krüger A., Calvani E., Michel S., Börno S., Christen S., Patil K.R., Timmermann B., Lilley K.S., Ralser M. (2016). The metabolic background is a global player in Saccharomyces gene expression epistasis. Nature Microbiology.

[bib3] Alter T.B., Ebert B.E. (2019). Determination of growth-coupling strategies and their underlying principles. BMC Bioinf..

[bib4] Annaluru N., Muller H., Mitchell L.A., Ramalingam S., Stracquadanio G., Richardson S.M., Dymond J.S., Kuang Z., Scheifele L.Z., Cooper E.M., Cai Y., Zeller K., Agmon N., Han J.S., Hadjithomas M., Tullman J., Caravelli K., Cirelli K., Guo Z., London V., Yeluru A., Murugan S., Kandavelou K., Agier N., Fischer G., Yang K., Martin J.A., Bilgel M., Bohutski P., Boulier K.M., Capaldo B.J., Chang J., Charoen K., Choi W.J., Deng P., DiCarlo J.E., Doong J., Dunn J., Feinberg J.I., Fernandez C., Floria C.E., Gladowski D., Hadidi P., Ishizuka I., Jabbari J., Lau C.Y., Lee P.A., Li S., Lin D., Linder M.E., Ling J., Liu J., Liu J., London M., Ma H., Mao J., McDade J.E., McMillan A., Moore A.M., Oh W.C., Ouyang Y., Patel R., Paul M., Paulsen L.C., Qiu J., Rhee A., Rubashkin M.G., Soh I.Y., Sotuyo N.E., Srinivas V., Suarez A., Wong A., Wong R., Xie W.R., Xu Y., Yu A.T., Koszul R., Bader J.S., Boeke J.D., Chandrasegaran S. (2014). Total synthesis of a functional designer eukaryotic chromosome. Science.

[bib5] Barton N.H. (2017). How does epistasis influence the response to selection?. Heredity.

[bib6] Carpenter A.C., Paulsen I.T., Williams T.C. (2018). Blueprints for biosensors: design, limitations, and applications. Genes.

[bib7] Caspeta L., Chen Y., Ghiaci P., Feizi A., Buskov S., Hallström B.M., Petranovic D., Nielsen J. (2014). Altered sterol composition renders yeast thermotolerant. Science.

[bib8] Chiotti K.E., Kvitek D.J., Schmidt K.H., Koniges G., Schwartz K., Donckels E.A., Rosenzweig F., Sherlock G. (2014). The Valley-of-Death: reciprocal sign epistasis constrains adaptive trajectories in a constant, nutrient limiting environment. Genomics.

[bib9] Chou H.H., Keasling J.D. (2013). Programming adaptive control to evolve increased metabolite production. Nat. Commun..

[bib10] Dawid A., Kiviet D.J., Kogenaru M., de Vos M., Tans S.J. (2010). Multiple peaks and reciprocal sign epistasis in an empirically determined genotype-phenotype landscape. Chaos.

[bib11] de Vos M.G.J., Dawid A., Sunderlikova V., Tans S.J. (2015).

[bib12] Dietrich J.A., Shis D.L., Alikhani A., Keasling J.D. (2013). Transcription factor-based screens and synthetic selections for microbial small-molecule biosynthesis. ACS Synth. Biol..

[bib13] Dragosits M., Mattanovich D. (2013). Adaptive laboratory evolution – principles and applications for biotechnology. Microb. Cell Factories.

[bib14] Espinosa M.I., Gonzalez-Garcia R.A., Valgepea K., Plan M.R., Scott C., Pretorius I.S., Marcellin E., Paulsen I.T., Williams T.C. (2020). Adaptive laboratory evolution of native methanol assimilation in Saccharomyces cerevisiae. Nat. Commun..

[bib15] Flynn K.M., Cooper T.F., Moore F.B.G., Cooper V.S. (2013). The environment affects epistatic interactions to alter the topology of an empirical fitness landscape. PLoS Genet..

[bib16] Hadany L. (2003). Adaptive peak shifts in a heterogenous environment. Theor. Popul. Biol..

[bib17] Hall A.E., Karkare K., Cooper V.S., Bank C., Cooper T.F., Moore F.B.-G. (2019). Environment changes epistasis to alter trade-offs along alternative evolutionary paths.

[bib18] Heckmann D., Schulze S., Denton A., Gowik U., Westhoff P., Weber A.P., Lercher M.J. (2013). Predicting C4 photosynthesis evolution: modular, individually adaptive steps on a Mount Fuji fitness landscape. Cell.

[bib19] Ho P.-W., Swinnen S., Duitama J., Nevoigt E. (2017). The sole introduction of two single-point mutations establishes glycerol utilization in Saccharomyces cerevisiae CEN.PK derivatives. Biotechnol. Biofuels.

[bib20] Hubbarde J.E., Wahl L.M. (2008). Estimating the optimal bottleneck ratio for experimental evolution: the burst-death model. Math. Biosci..

[bib21] Iwasa Y., Michor F., Nowak M.A. (2004). Stochastic tunnels in evolutionary dynamics. Genetics.

[bib22] Jensen E.D., Laloux M., Lehka B.J., Pedersen L.E., Jakočiūnas T., Jensen M.K., Keasling J.D. (2021). A synthetic RNA-mediated evolution system in yeast. Nucleic Acids Res..

[bib23] Jha R.K., Kern T.L., Fox D.T., Strauss C.E M. (2014). Engineering an Acinetobacter regulon for biosensing and high-throughput enzyme screening in E. coli via flow cytometry. Nucleic Acids Res..

[bib24] Kashtan N., Noor E., Alon U. (2007). Varying environments can speed up evolution. Proc. Natl. Acad. Sci. U. S. A..

[bib25] Kvitek D.J., Sherlock G. (2011). Reciprocal sign epistasis between frequently experimentally evolved adaptive mutations causes a rugged fitness landscape. PLoS Genet..

[bib26] LaCroix R.A., Palsson B.O., Feist A.M. (2017). A model for designing adaptive laboratory evolution experiments. Appl. Environ. Microbiol..

[bib27] Lee D.-H., Feist A.M., Barrett C.L., Palsson B.Ø. (2011). Cumulative number of cell divisions as a meaningful timescale for adaptive laboratory evolution of Escherichia coli. PLoS One.

[bib28] Liu Y.n., Li Q., Zheng P., Zhang Z., Liu Y., Sun C., Cao G., Zhou W., Wang X., Zhang D., Zhang T., Sun J., Ma Y. (2015). Developing a high-throughput screening methodfor threonine overproduction based on an artificial promoter. Microb. Cell Factories.

[bib29] Liu Y., Zhuang Y., Ding D., Xu Y., Sun J., Zhang D. (2017). Biosensor-based evolution and elucidation of a biosynthetic pathway in Escherichia coli. ACS Synth. Biol..

[bib30] Maltas J., McNally D.M., Wood K.B. (2021). Evolution in alternating environments with tunable interlandscape correlations. Evolution.

[bib31] Mans R., Daran J.-M.G., Pronk J.T. (2018). Under pressure: evolutionary engineering of yeast strains for improved performance in fuels and chemicals production. Curr. Opin. Biotechnol..

[bib32] Marcellin E., Nielsen L.K. (2018). Advances in analytical tools for high throughput strain engineering. Curr. Opin. Biotechnol..

[bib33] Meyer F., Keller P., Hartl J., Gröninger O.G., Kiefer P., Vorholt J.A. (2018). Methanol-essential growth of Escherichia coli. Nat. Commun..

[bib34] Nielsen J., Keasling Jay D. (2016). Engineering cellular metabolism. Cell.

[bib35] Obolski U., Ram Y., Hadany L. (2017). Key issues review: evolution on rugged adaptive landscapes. Rep. Prog. Phys..

[bib36] Ochs I.E., Desai M.M. (2015). The competition between simple and complex evolutionary trajectories in asexual populations. BMC Evol. Biol..

[bib37] Pál C., Papp B. (2017). Evolution of complex adaptations in molecular systems. Nature Ecology & Evolution.

[bib38] Poelwijk F.J., Kiviet D.J., Weinreich D.M., Tans S.J. (2007). Empirical fitness landscapes reveal accessible evolutionary paths. Nature.

[bib39] Poelwijk F.J., Tănase-Nicola S., Kiviet D.J., Tans S.J. (2011). Reciprocal sign epistasis is a necessary condition for multi-peaked fitness landscapes. J. Theor. Biol..

[bib40] Portnoy V.A., Bezdan D., Zengler K. (2011). Adaptive laboratory evolution—harnessing the power of biology for metabolic engineering. Curr. Opin. Biotechnol..

[bib41] Reetz M.T., Sanchis J. (2008). Constructing and analyzing the fitness landscape of an experimental evolutionary process. Chembiochem.

[bib42] Rix G., Watkins-Dulaney E.J., Almhjell P.J., Boville C.E., Arnold F.H., Liu C.C. (2020). Scalable continuous evolution for the generation of diverse enzyme variants encompassing promiscuous activities. Nat. Commun..

[bib43] Romero P.A., Arnold F.H. (2009). Exploring protein fitness landscapes by directed evolution. Nat. Rev. Mol. Cell Biol..

[bib44] Sailer Z.R., Harms M.J. (2017). High-order epistasis shapes evolutionary trajectories. PLoS Comput. Biol..

[bib45] Sandberg T.E., Lloyd C.J., Palsson B.O., Feist A.M. (2017). Laboratory evolution to alternating substrate environments yields distinct phenotypic and genetic adaptive strategies. Appl. Environ. Microbiol..

[bib46] Sandberg T.E., Salazar M.J., Weng L.L., Palsson B.O., Feist A.M. (2019). The emergence of adaptive laboratory evolution as an efficient tool for biological discovery and industrial biotechnology. Metab. Eng..

[bib47] Sato T.K., Tremaine M., Parreiras L.S., Hebert A.S., Myers K.S., Higbee A.J., Sardi M., McIlwain S.J., Ong I.M., Breuer R.J., Avanasi Narasimhan R., McGee M.A., Dickinson Q., La Reau A., Xie D., Tian M., Reed J.L., Zhang Y., Coon J.J., Hittinger C.T., Gasch A.P., Landick R. (2016). Directed evolution reveals unexpected epistatic interactions that alter metabolic regulation and enable anaerobic xylose use by Saccharomyces cerevisiae. PLoS Genet..

[bib48] Schallmey M., Frunzke J., Eggeling L., Marienhagen J. (2014). Looking for the pick of the bunch: high-throughput screening of producing microorganisms with biosensors. Curr. Opin. Biotechnol..

[bib49] Sciambi A., Abate A.R. (2015). Accurate microfluidic sorting of droplets at 30 kHz. Lab Chip.

[bib50] Shepelin D., Hansen A.S.L., Lennen R., Luo H., Herrgard M.J. (2018). Selecting the best: evolutionary engineering of chemical production in microbes. Genes.

[bib51] Snitkin E.S., Segrè D. (2011). Epistatic interaction maps relative to multiple metabolic phenotypes. PLoS Genet..

[bib52] Steinberg B., Ostermeier M. (2016). Environmental changes bridge evolutionary valleys. Sci. Adv..

[bib53] Sugrue E., Scott C., Jackson C.J. (2017). Constrained evolution of a bispecific enzyme: lessons for biocatalyst design. Org. Biomol. Chem..

[bib54] Szappanos B., Fritzemeier J., Csörgő B., Lázár V., Lu X., Fekete G., Bálint B., Herczeg R., Nagy I., Notebaart R.A., Lercher M.J., Pál C., Papp B. (2016). Adaptive evolution of complex innovations through stepwise metabolic niche expansion. Nat. Commun..

[bib55] Szendro I.G., Schenk M.F., Franke J., Krug J., de Visser J.A.G.M. (2013). Quantitative analyses of empirical fitness landscapes. J. Stat. Mech. Theor. Exp..

[bib56] Taute K.M., Gude S., Nghe P., Tans S.J. (2014). Evolutionary constraints in variable environments, from proteins to networks. Trends Genet..

[bib57] Tracewell C.A., Arnold F.H. (2009). Directed enzyme evolution: climbing fitness peaks one amino acid at a time. Curr. Opin. Chem. Biol..

[bib58] Van Cleve J., Weissman D.B. (2015). Measuring ruggedness in fitness landscapes. Proc. Natl. Acad. Sci. U. S. A..

[bib59] Voordeckers K., Kominek J., Das A., Espinosa-Cantú A., De Maeyer D., Arslan A., Van Pee M., van der Zande E., Meert W., Yang Y., Zhu B., Marchal K., DeLuna A., Van Noort V., Jelier R., Verstrepen K.J. (2015). Adaptation to high ethanol reveals complex evolutionary pathways. PLoS Genet..

[bib60] Wahl L.M., Gerrish P.J. (2001). The probability that beneficial mutations are lost in populations with periodic bottlenecks. Evolution.

[bib61] Wang H.H., Isaacs F.J., Carr P.A., Sun Z.Z., Xu G., Forest C.R., Church G.M. (2009). Programming cells by multiplex genome engineering and accelerated evolution. Nature.

[bib62] Weinreich D.M., Watson R.A., Chao L. (2005). PERSPECTIVE: sign epistasis and genetic COSTRAINT on evolutionary trajectories. Evolution.

[bib63] Weinreich D.M., Lan Y., Wylie C.S., Heckendorn R.B. (2013). Should evolutionary geneticists worry about higher-order epistasis?. Curr. Opin. Genet. Dev..

[bib64] Weissman D.B., Desai M.M., Fisher D.S., Feldman M.W. (2009). The rate at which asexual populations cross fitness valleys. Theor. Popul. Biol..

[bib65] Williams T.C., Pretorius I.S., Paulsen I.T. (2016). Synthetic evolution of metabolic productivity using biosensors. Trends Biotechnol..

[bib66] Wright S. (1932).

[bib67] Wu Y., Zhu R.-Y., Mitchell L.A., Ma L., Liu R., Zhao M., Jia B., Xu H., Li Y.-X., Yang Z.-M., Ma Y., Li X., Liu H., Liu D., Xiao W.-H., Zhou X., Li B.-Z., Yuan Y.-J., Boeke J.D. (2018). In vitro DNA SCRaMbLE. Nat. Commun..

[bib68] Xu X., Williams T.C., Divne C., Pretorius I.S., Paulsen I.T. (2019). Evolutionary engineering in Saccharomyces cerevisiae reveals a TRK1-dependent potassium influx mechanism for propionic acid tolerance. Biotechnol. Biofuels.

[bib69] Yang G., Anderson D.W., Baier F., Dohmen E., Hong N., Carr P.D., Kamerlin S.C.L., Jackson C.J., Bornberg-Bauer E., Tokuriki N. (2019). Higher-order epistasis shapes the fitness landscape of a xenobiotic-degrading enzyme. Nat. Chem. Biol..

[bib70] Zheng Y., Hong K., Wang B., Liu D., Chen T., Wang Z. (2021). Genetic diversity for accelerating microbial adaptive laboratory evolution. ACS Synth. Biol..

